# BMC ear, nose and throat disorders reviewer acknowledgment 2015

**DOI:** 10.1186/s12901-016-0023-7

**Published:** 2016-03-10

**Authors:** Elaine Zhang

**Affiliations:** BMC Ear, nose and throat disorders, BioMed Central, 233 Spring Street, New York, NY 10013 USA

## Abstract

The editors of BMC Ear, Nose and Throat Disorders would like to thank all our reviewers who have contributed to the journal in Volume 15 (2015).

**Abraham Alabi**

Gabon

**Stephen Ball**

UK

**Duncan Bowyer**

France

**Jean-Francois Chenot**

Germany

**Paul Counter**

UK

**Robin Criter**

USA

**Osama Dessouky**

UK

**Richard Dowell**

Australia

**Ronald Eccles**

UK

**Thorlene Egerton**

Norway

**Gregory Grillone**

USA

**Hasantha Gunasekera**

Australia

**Hiroaki Ichijo**

Japan

**Steve Izzat**

UK

**Sue Jones**

UK

**Seung-Han Lee**

Korea, South

**Avril Mansfield**

Canada

**Jan Matthys**

Belgium

**Omar Mirza**

UK

**Oke Okonkwo**

UK

**Okechukwu Okonkwo**

UK

**Folu Ologe**

Nigeria

**Bolajoko O. Olusanya**

Nigeria

**Vijay Pothula**

UK

**James Ramsden**

UK

**Baskaran Ranganathan**

UK

**Julie Schneider**

Australia

**Laura Tod**

UK

**Matthew Trotter**

France

**Laura Warner**

UK

**Brian Westerberg**

Canada

